# Hair Toxic Metal Concentrations and Autism Spectrum Disorder Severity in Young Children

**DOI:** 10.3390/ijerph9124486

**Published:** 2012-12-06

**Authors:** David A. Geier, Janet K. Kern, Paul G. King, Lisa K. Sykes, Mark R. Geier

**Affiliations:** 1 Institute of Chronic Illnesses, 14 Redgate Ct, Silver Spring, MD 20905, USA; E-Mail: davidallengeier@comcast.net.net; 2 University of Texas Southwestern Medical Center at Dallas, 5353 Harry Hine Blvd, Dallas, TX 75390, USA; E-Mail: jkern@dfwair.net; 3 CoMeD, Inc., 14 Redgate Ct, Silver Spring, MD 20905, USA; E-Mails: paulgkingphd@gmail.com (P.G.K.); syklone5@verizon.net (L.K.S.)

**Keywords:** asperger, autism, autistic, developmental, hair, heavy metal, neurodevelopmental, neurotoxicity, PDD-NOS

## Abstract

Previous studies have found a higher body-burden of toxic metals, particularly mercury (Hg), among subjects diagnosed with an autism spectrum disorder (ASD) in comparison to neurotypical controls. Moreover, Hg body-burden was associated with ASD severity. This cross-sectional study examined the potential correlation between hair toxic metal concentrations and ASD severity in a prospective cohort of participants diagnosed with moderate to severe ASD. The Institutional Review Board at the University of Texas Southwestern Medical Center at Dallas (Dallas, TX) approved the present study. Qualifying study participants (n = 18) were evaluated for ASD severity using the Childhood Autism Rating Scale (CARS) and quantitatively for arsenic, Hg, cadmium, lead, chromium, cobalt, nickel, aluminum, tin, uranium, and manganese using hair toxic element testing by Doctor’s Data (a CLIA-approved laboratory). CARS scoring and hair toxic element testing were blinded to one another. Increasing hair Hg concentrations significantly correlated with increased ASD severity. In contrast, no significant correlations were observed between any other of the hair toxic metals examined and ASD severity. This study helps to provide additional mechanistic support for Hg in the etiology of ASD severity, and is supported by an increasing number of recent critical reviews that provide biological plausibility for the role of Hg exposure in the pathogenesis of ASDs.

## 1. Introduction

Desoto and Hitlan undertook a review of published research studies examining the relationship between toxic metal exposures and the risk of a subject being diagnosed with an autism spectrum disorder (ASD) [1]. These investigators identified 58 research articles which provided empirical evidence relevant to the question of a link between an ASD diagnosis and one or more toxic metal exposures. Of those 58 research articles examined, these investigators identified 43 that supported a significant link between an ASD diagnosis and exposure to toxic metals while 15 showed no statistically significant evidence of a link between an ASD diagnosis and exposure to toxic metals. Thus, 74% of the studies examined showed a significant relationship between an ASD diagnosis and toxic metal exposure. These investigators concluded that the balance of studies support a link between ASD diagnoses and toxic metal exposure.

Since Desoto and Hitlan’s review, more studies have shown a higher body burden of toxic metals in subjects diagnosed with an ASD compared to neurotypical controls [2,3,4,5,6,7,8,9] and have also revealed a significant relationship between toxic metal body-burden and ASD severity [5,7,10,11,12,13]. Several recent studies have also failed to find a relationship between a higher body burden of toxic metals and an ASD diagnosis [14,15,16,17]. Although studies have shown an association between an ASD diagnosis and various toxic metals, such as cadmium (Cd), lead (Pb), and arsenic (As), the bulk of the research has focused on mercury (Hg).

For example, Lakshmi *et al*. assessed toxic elements such as Hg and Pb in the hair and nail samples of subjects diagnosed with an ASD and evaluated whether the level of these elements could be correlated with the severity of an ASD diagnosis [7]. The study showed a significant elevation in the concentrations of toxic metals Pb and Hg in both the hair and nail samples of those subjects diagnosed with an ASD when compared to neurotypical controls. The elevation was much pronounced in low functioning participants as opposed to moderate to high functioning participants. In addition, a study by Elsheshtawy *et al*. revealed similar findings [5]. They found highly significant differences between the level of Pb and Hg in the hair of subjects diagnosed with autism compared to neurotypical controls. These investigators observed a significant correlation between increasing ASD severity measured by the Childhood Autism Rating Scale (CARS) and increasing Hg in the hair of study subjects diagnosed with an ASD, whereas no significant correlation was observed between increasing ASD severity and increasing Pb in the hair of study subjects.

Furthermore, recent studies suggest that the impact of toxic metals may be more evident in subjects diagnosed with moderate to severe ASD as opposed to participants diagnosed with a mild ASD [12,13]. It was even observed that toxic metal excretion pathways may significantly vary among study subjects diagnosed with moderate to severe ASD as opposed to participants diagnosed with a mild ASD [10,18]. This may be of particular importance when examining hair toxic metal concentrations in young children because previous studies have suggested that hair toxic concentrations may be related to toxic metal excretion rates, especially in first baby-haircuts of subjects diagnosed with an ASD [3,19,20,21]. Hence, differences in excretion may introduce a confounding variable when examining the correlation between toxic metal in hair samples and ASD severity.

The purpose of the present study was to evaluate the potential correlation between concentrations of hair toxic metals and a quantitative measure of ASD severity in a prospectively collected cohort of participants diagnosed with moderate to severe ASD.

## 2. Experimental Section

### 2.1. Institutional Review Board Approval and Human Participants Compliance

The protocol employed in the present study received approval from the Institutional Review Board of the University of Texas Southwestern Medical Center at Dallas (Dallas, TX, USA). The study complied with the American Psychological Association ethical standards in the treatment of participants and in obtaining informed consent. All parents signed a consent and Health Insurance Portability and Accountability Act (HIPAA) form and all received a copy of both forms. Children were in the presence of one or both parents throughout the assessment visit.

### 2.2. Participants

Data were collected from children that reside in the Dallas Metropolitan area and surrounding communities. Participants were included in the present study if they had a previous ASD diagnosis, which was confirmed at the time of the study by the principal investigator (JKK) based on the *Diagnostic and Statistical Manual of Mental Disorders*, Fourth Edition (DSM-IV) criteria [22] and CARS evaluation determined by the principal investigator (JKK) [23,24]. Participants had to have moderate to severe CARS scores (37–60) to be included in the present study. The data examined were collected prospectively from consecutive children that met the study entrance criteria.

None of the children had been or were currently receiving chelation treatment. None of the children were on any diets or supplements that were out of the ordinary. None of the children had Fragile X disorder, tuberous sclerosis, phenylketonuria (PKU), Lesch-Nyhan syndrome, fetal alcohol syndrome, or history of maternal drug use. Table 1 summarizes demographic information collected on each subject.

### 2.3. Measurements

The CARS is a 15-item behavioral rating scale developed to identify autism as well as to quantitatively describe the severity of the disorder. A total score of 15–29.5 is considered nonautistic; a score of 30–36.5 is considered mild to moderate autism; a score from 37–60 is considered moderate to severe autism. The CARS is well-established measure. The internal consistency reliability α-coefficient is 0.94; the inter-rater reliability correlation coefficient is 0.71; and the test-retest correlation coefficient is 0.88. CARS scores have high criterion-related validity when compared to clinical ratings during the same diagnostic sessions, with a significant correlation of 0.84 [25]. The study principal investigator (JKK) has had significant experience in evaluating many study participants diagnosed with an ASD using CARS scoring. Table 1 summarizes the overall mean CARS scores ± standard deviation for the participants studied.

**Table 1:** A demographic summary of the participants diagnosed with an ASD examined.

Descriptive Information	Summary (n = 18)
**Sex*/*Age**	
Male*/*Female (ratio)	15*/*3 (5:1)
Mean Age in Years ± Std (range)	3.5 ± 1.1 (1–6)
Mean Year of Birth ± Std (range)	2001 ± 1.1 (1999–2003)
**Race (n)**	
Caucasian	72% (13)
Minorities ^1^	28% (5)
**Autistic Disorder Characteristics**	
Mean CARS Score ± Std (range)	41.4 ± 4.1 (37–51)
Non-Regressive (n)	44.4% (8)
Regressive (n) ^2^	55.6% (10)

CARS = Childhood Autism Rating Scale; ^1 ^Includes participants of Hispanic, Black, Asian, or Mixed Ancestry; ^2 ^Includes participants that had a regressive event in development at any time following birth.

Blinded hair toxic element exposure profile testing was conducted by Doctor’s Data, a Clinical Laboratory Improvement Act/Amendment (CLIA)-approved laboratory, on each study participant using a uniform protocol. To minimize hair care variance, all study participants had their hair washed with Johnson & Johnson’s baby shampoo for at least one week prior to collection of hair samples. All hair specimens were collected as instructed by Doctor’s Data using stainless steel scissors, and were cut from hair within 2 inches of the scalp (to reduce exposure to external contaminants). Hair samples were mailed to Doctor’s Data in the individual kits provided and analyzed using their laboratory analysis protocols [26,27]. At Doctor’s Data, the hair specimens were further cut and washed using a modified method developed by the International Atomic Energy Agency. The hair specimens were cut into approximately 0.3 cm pieces and mixed to allow a representative sub-sampling of the hair specimen. After cutting, each sample was washed four times with a 1:200 v/v dilution of Triton X-100, then rinsed with acetone and allowed to drain. Samples were then rinsed three times with ultra-pure deionized water and two times with acetone. The dried samples were weighed prior to nitric acid/microwave digestion. After digestion, the samples were cooled and a 500 µL aliquot of an internal standard (IS) was added. Each of the resultant IS-spiked samples were diluted with 50 mL of ultrapure, deionized water. The individual diluted samples were then analyzed for element content using Inductively Coupled Plasma-Mass Spectrometry (ICP-MS). To ensure validity, the following were analyzed by Doctor’s Data: calibration verification standards, a certified hair reference control, in-house controls, spiked hair samples and other appropriate control samples were analyzed by Doctor’s Data [26,27]. The present study reports on hair toxic metal results expressed as µg toxic metal per g hair for As, Pb, Hg, Cd, chromium (Cr), cobalt (Co), nickel (Ni), manganese (Mn), aluminum (Al), tin (Sn), and uranium (U). Table 2 summarizes the overall mean ± standard deviation hair concentrations for each toxic metal examined in the present study.

**Table 2:** A summary of hair toxic metal concentrations among participants diagnosed with an ASD examined.

Hair Metal Type	Mean Level ^1^ ± Std (range)
Arsenic (As)	0.074 ± 0.058 (0.02–0.26)
Lead (Pb)	0.63 ± 0.50 (0.18–1.7)
Mercury (Hg)	0.33 ± 1.01 (0.03–4.4)
Cadmium (Cd)	0.18 ± 0.14 (0.054–0.53)
Chromium (Cr)	0.41 ± 0.11 (0.29–0.69)
Cobalt (Co)	0.03 ± 0.03 (0.003 ± 0.15)
Nickel (Ni)	0.17 ± 0.1 (0.05–0.46)
Manganese (Mn)	0.4 ± 0.4 (0.08–1.7)
Aluminum (Al)	13 ± 5.8 (6.2–29)
Tin (Sn)	0.44 ± 0.25 (0.12–0.96)
Uranium (U)	0.046 ± 0.035 (0.003–0.13)

^1^ µg of metal per g of hair.

### 2.4. Study Design

The CARS was completed on all of the study participants by the study principal investigator (JKK), who observed the participants and interviewed their parent(s). Subsequent to the initial evaluation of each subject by the study principal investigator (JKK), hair was collected on each subject for the hair toxic elements exposure profile testing, and the hair was mailed to Doctor’s Data laboratory in the individual test kits provided. Each specimen submitted to the Doctor’s Data laboratory was encoded, and the laboratory received no clinical information about participants providing samples for analysis.

### 2.5. Statistical Analysis

The statistical package contained in StatsDirect (version 2.7.8) was utilized for all statistical testing, and a two-sided *p*-value < 0.05 was considered statistically significant in the present study. In order to evaluate the potential relationship between ASD severity and hair toxic metals (n = 18), the Spearman’s rank correlation test statistic was utilized. The results of the analysis determined the Spearman’s rank correlation (Rho), 95% confidence interval for Rho (Fisher’s Z transformed), and a two-sided *p*-value. The null hypothesis was that no relationship between ASD severity and hair toxic metal concentrations existed. The Spearman’s rank correlation test statistic was utilized in the present study because it is generally recognized as a nonparametric statistical test, and hence, it requires minimal assumptions regarding the overall distribution of the data examined.

## 3. Results and Discussion

As summarized in Table 1, the study participants examined were between 1 to 6 years-old with a mean age of 3.5 ± 1.1 years-old. There were more male than female participants examined (male/female ratio = 5:1). Overall, the mean year of birth was 2001 ± 1.1 (range = 1999 to 2003). Among study participants, Caucasians (72%) were more preponderant than minorities (28%). The overall mean CARS score was 41.4 ± 4.1 (range = 37 to 51) for study participants. Further, more of the study participants had experienced a regressive event in development some time following birth (55.6%) than had not (44.4%).

Table 3 summarizes the correlation between hair toxic metal concentrations and ASD severity as established by the Spearman’s rank correlation test statistic. Increasing hair Hg concentrations were found to significantly correlate with increasing ASD severity (Rho = 0.58, *p* = 0.013). Further, removing the anomalous highest Hg data point (CARS score = 43, hair Hg level = 4.4 µg of Hg per g of hair) from the sample set being evaluated still revealed a significant correlation (Rho = 0.57, *p* = 0.018). In contrast, no significant correlations were observed between any of the other hair toxic metal concentrations and ASD severity.

**Table 3:** A summary of the correlation^1^ between hair toxic metals concentrations and ASD severity^2^.

Hair Metal Type	Rho	Rho 95% Confidence Interval	*p*-value
Arsenic (As)	−0.13	−0.56 to 0.36	0.62
Lead (Pb)	−0.31	−0.68 to 0.18	0.21
Mercury (Hg)	0.58	0.15 to 0.82	0.013 ^3^
Cadmium (Cd)	−0.33	−0.69 to 0.16	0.18
Chromium (Cr)	0.30	−0.19 to 0.67	0.22
Cobalt (Co)	−0.34	−0.69 to 0.15	0.17
Nickel (Ni)	−0.10	−0.54 to 0.39	0.70
Manganese (Mn)	−0.11	−0.55 to 0.37	0.65
Aluminum (Al)	0.08	−0.40 to 0.53	0.75
Tin (Sn)	−0.14	−0.57 to 0.35	0.59
Uranium (U)	0.23	−0.27 to 0.63	0.36

^1 ^The Spearman’s rank correlation test statistic was utilized; ^2 ^ASD severity was measured using the Childhood Autism Rating Scale (CARS) score; ^3 ^Eliminating the anomalous highest hair Hg data point (CARS score = 43, hair mercury level = 4.4 µg of Hg per g of hair) from the sample set examined still revealed a significant correlation (Rho = 0.57, Rho 95% confidence interval = 0.12 to 0.82, *p* = 0.018) between hair Hg concentrations and ASD severity.

**Figure 1:**
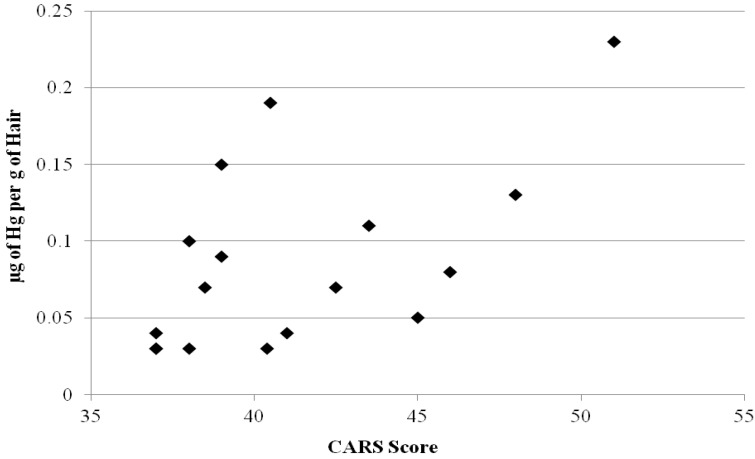
A summary of the scatter plot distribution of hair Hg concentrations in comparison to CARS scores. The scatter plot distribution excludes the highest anomalous hair Hg observation.

Figure 1 summarizes the scatter plot distribution of hair Hg concentrations in comparison to CARS scores. The results of the present study suggest a significant correlation between increasing ASD severity and increasing hair Hg concentrations among study participants diagnosed with a moderate to severe ASD. In contrast, the other toxic metals showed no significant association with ASD severity. As a result, the present study helps to provide additional mechanistic support for Hg in the etiology of the clinical severity of an ASD diagnosis. The results observed in the present study are supported by an increasing number of recent critical reviews that provide biological plausibility for Hg exposure playing a significant etiological role in the pathogenesis of ASDs.

For example, Garrecht and Austin reported that Hg is recognized as a ubiquitous environmental neurotoxin and that there is mounting evidence linking it to ASDs from methods focusing on biomarkers of Hg damage, measurements of Hg exposure, epidemiological data, and animal studies [28]. Among the key areas shown to associate Hg exposure with an ASD diagnosis examined by these investigators are: (1) the route and cellular mechanisms of Hg exposure in ASDs; (2) examples of possible genetic variables that are linked to both Hg sensitivity and ASDs; (3) the role Hg may play as an environmental toxin fueling the oxidative stress found in ASDs; (4) the role of mitochondrial dysfunction; and (5) the role of Hg in abnormal neuroexcitory and excitotoxity that may play a role in the immune dysregulation found in those with an ASD diagnosis.

Similarly, investigators described that Hg is widespread and persistent in the environment [29,30,31]. Hg is a ubiquitous source of danger in fish, drugs, fungicides/herbicides, dental fillings, thermometers, vaccines, and many other products/food stuffs. Elevated Hg concentrations may remain in the brain from several years to decades following exposure. This is important because investigators have long recognized that Hg is a bio-accumulative neurodevelopmental poison; it can cause problems in neuronal cell migration and division, and can ultimately cause cell degeneration and death. Case-reports of subjects have described developmental regressions with ASD symptoms following fetal and/or early childhood Hg exposure, and epidemiological studies have linked exposure to Hg with an elevated risk of a subject being diagnosed with an ASD. Immune, sensory, neurological, motor, and behavioral dysfunctions similar to traits defining or associated with ASDs have been reported following Hg intoxication with similarities extending to neuroanatomy, neurotransmitters, and biochemistry.

Finally, Kern *et al*. examined the parallels between the effects Hg intoxication on the brain and the brain pathology found in those diagnosed with an ASD [32]. These investigators found evidence of many parallels between the two, including: (1) microtubule degeneration, specifically large, long-range axon degeneration with subsequent abortive axonal sprouting (short, thin axons); (2) dentritic overgrowth; (3) neuroinflammation; (4) microglial/astrocytic activation; (5) brain immune response activation; (6) elevated glial fibrillary acidic protein; (7) oxidative stress and lipid peroxidation; (8) decreased reduced glutathione levels and elevated oxidized glutathione; (9) mitochondrial dysfunction; (10) disruption in calcium homeostasis and signaling; (11) inhibition of glutamic acid decarboxylase (GAD) activity; (12) disruption of GABAergic and glutamatergic homeostasis; (13) inhibition of IGF-1 and methionine synthase activity; (14) impairment in methylation; (15) vascular endothelial cell dysfunction and pathological changes of the blood vessels; (16) decreased cerebral/cerebellar blood flow; (17) increased amyloid precursor protein; (18) loss of granule and Purkinje neurons in the cerebellum; (19) increased pro-inflammatory cytokine levels in the brain (TNF-α, IFN-γ, IL-1β, IL-8); and (20) aberrant nuclear factor kappa-light-chain-enhancer of activated B cells (NF-kappaB). These investigators also examined the ability of Hg to potentiate and work synergistically with other toxins and pathogens in a way that may contribute to the brain pathology in ASD. These investigators concluded that the evidence suggests that Hg may be either causal or contributory in the brain pathology in ASD, possibly working synergistically with other toxic compounds or pathogens, to produce the brain pathology observed in those diagnosed with an ASD.

### 3.1. Study Strengths

Key among the strengths of the present study is the correspondence of its results to those reported by previous investigators. This is especially true with respect to a previous study by Elsheshtawy *et al*. [5]. These investigators examined a cohort of study subjects diagnosed with an ASD of similar age and sex distribution as those examined in the present study. These investigators observed a significant correlation between increasing ASD severity measured by CARS and increasing Hg in the hair of study subjects, comparable to the results observed in the present study. Just as in the present study, these investigators failed to find a significant relationship between Pb hair concentrations and ASD severity measured by CARS. They even observed, consistent with the present study, a non-significant trend toward a correlation between increasing Pb hair concentrations and decreasing ASD severity measured by CARS.

Another significant strength of the present study is that only study participants with an ASD diagnosis of moderate to severe severity were included. This strength of the present study occurred because previous studies suggested first that there might be differences in toxic metal hair excretion patterns among subjects diagnosed with an ASD in comparison to neurotypical controls [3,19,20,21]. Secondly, previous studies also suggested that even among subjects diagnosed with an ASD, it had been observed previously observed that study subjects diagnosed moderate to severe ASD severity had significantly different concentrations of toxic metals than study subjects diagnosed with an ASD of mild severity [12,13]. As a result, the aforementioned concerns did not need to be considered in the present study because all of the study participants examined were diagnosed with a moderate to severe ASD. In addition, the present study utilized an *a priori* definition of moderate to severe ASD severity for CARS scores (37 to 60) derived from previously published investigations, so that no post data collection biases could be introduced by the present investigators in regard to methods of defining ASD severity.

### 3.2. Study Limitations

In considering the limitations of the present study, the sample of study participants examined was small. As a result, it is possible that with additional numbers of study participants, the study design employed would have had additional statistical power to detect potential associations between hair toxic metal concentrations and ASD severity measured by CARS. Despite this potential limitation, the present study did have adequate statistical power to detect a significant association between Hg hair concentrations and ASD severity. Another potential limitation of the present study was that some of the results observed may have occurred because of a statistical chance or because of some unknown bias in the data examined. This potential limitation should have had a small impact on the observations made because a limited number of statistical tests were conducted on the present data sample, and the size and specificity of the correlations observed argues against the results of this study being consequence of statistical chance or because of some unknown bias. Also, the statistical robustness of the present findings indicates a significant correlation between Hg hair concentrations and ASD severity, even after eliminating the anomalous highest hair Hg value from the results reported. This argues against attributing the results observed to statistical chance or some unknown bias. Further, the consistency of the results in the present study with previous studies additionally argues against statistical chance or unknown bias in the data. Another potential limitation of the present study was that CARS testing was utilized to help confirm an ASD diagnosis and measure overall ASD severity. It is possible that other tests such as Autism Diagnostic Observation Schedule (ADOS) or Autism Diagnostic Interview, Revised (ADI-R) may have yielded different results than those observed in the present study. It would be of use in future studies to employ other ASD tests to determine the comparability of results obtained with those found in the present study. It is also not possible with the prospective cross-sectional ASD cohort design of the present study to assign a causal relationship between Hg exposure and ASD severity or to draw conclusions between Hg concentrations in subjects diagnosed with an ASD in comparison to neurotypical controls. Despite these limitations, the significant relationship between increasing hair Hg concentrations and ASD severity argues that the Hg concentrations did have a significant impact on ASD severity. Therefore, future studies should evaluate the potential etiological basis of Hg exposure with ASD severity in different populations. Finally, the present study also did not allow us to determine the sources of Hg significantly contributing to hair Hg concentrations observed. Interestingly, investigators in a previous small study published on hair Hg concentrations in children diagnosed with an ASD, with similar demographics to the participants examined in the present study, observed that Hg exposure from vaccines containing Thimerosal significantly contributed to hair Hg concentrations, where as a mild non-significant increases of mean hair Hg concentrations were observed in subjects diagnosed with an ASD whose mothers had increasing numbers of dental amalgams during pregnancy or had increasing fish consumption during pregnancy [33]. As a result, future studies should evaluate the relationship between different sources of Hg exposure and hair Hg concentrations in subjects diagnosed with an ASD.

## 4. Conclusions

In conclusion, the present study provides additional evidence of a linkage between Hg and an ASD diagnosis. The results observed are consistent with previous studies, and help to reveal mechanistic insights into the relationship between increasing Hg concentrations and increasing ASD diagnosis severity. Future studies should further evaluate the relationship between Hg exposure and ASD diagnosis severity in other study populations.
